# Applicability of common inflammatory markers in diagnosing infections in early period after liver transplantation in intensive care setting

**DOI:** 10.1038/s41598-020-60936-0

**Published:** 2020-03-03

**Authors:** Wojciech Figiel, Michał Grąt, Grzegorz Niewiński, Waldemar Patkowski, Krzysztof Zieniewicz

**Affiliations:** 1 Department of General, Transplant and Liver Surgery, Medical University of Warsaw, Warsaw, Poland; 2 II Department of Anaesthesiology and Intensive Care, Medical University of Warsaw, Warsaw, Poland

**Keywords:** Predictive markers, Liver cirrhosis, Bacterial infection, Liver

## Abstract

Infections remain an important cause of morbidity and mortality early after liver transplantation. The aim of this prospective longitudinal study was to evaluate clinical utility of c-reactive protein (CRP), procalcitonin, and neutrophil-to-lymphocyte ratio (NLR) in surveillance of infections early after liver transplantation in intensive care setting. A total of 60 liver transplant recipients were included. CRP, procalcitonin, and NLR assessed at 12-hour intervals were primary variables of interest. Infections and severe complications during postoperative intensive care unit stay were the primary and secondary end-points, respectively. Infections and severe complications were diagnosed in 9 and 17 patients, respectively. Only peak CRP beyond first 48 hours was associated with infections (p = 0.038) with AUC, positive and negative predictive value of 0.728, 42.9% and 92.2%, respectively (cut-off: 142.7 mg/L). Peak procalcitonin over first 60 hours was the earliest predictor (p = 0.050) of severe complications with AUC, positive and negative predictive value of 0.640, 53.3% and 80.0%, respectively (cut-off: 42.8 ng/mL). In conclusion, while CRP, procalcitonin, and NLR cannot be used for accurate diagnosis of infections immediately after liver transplantation, peak CRP beyond 48 hours and peak procalcitonin over first 60 hours may be used for initial exclusion of infections and prediction of severe complications, respectively.

## Introduction

Despite remarkable improvement of outcomes of patients after liver transplantation over past decades, infections remain one of the most important threats and most common causes of death in a population of liver transplant recipients^1–3^. In the early post-transplant period, they are the most common cause of morbidity^4^. Even 70% of liver transplant recipients develop bacterial infections, with vast majority occurring during the first post-transplant month^5,6^. These complications remain an important cause of death in the early post-transplant period with overall infection-related mortality rates varying between 0–30% depending on etiology, timing, and site^7–10^. Particularly high mortality rates, in the range of approximately 35–70%, are reported for patients developing infections with multiple drug resistant microorganisms, such as extended spectrum beta-lactamase-producing or carbapenem-resistant *Enterobacteriaceae*^11–13^. Improvements in prevention, early diagnosis and management of infections in the early period after liver transplantation are thus undoubtedly necessary to further improve patient outcomes. Several preventive measures, such as perioperative use of probiotics or implementation of bundled strategies, were recently proven effective in major reduction of post-transplant burden of infections^14–16^. However, early diagnosis of infectious complications after liver transplantation, a prerequisite for timely initiation of antimicrobial therapy, remains a major clinical challenge, particularly in the immediate postoperative period^17^.

Serum procalcitonin and C-reactive protein (CRP) concentrations and neutrophil-to-lymphocyte ratio (NLR) are well-established markers of systemic inflammation utilized for diagnosing postoperative infectious episodes in a non-transplant setting, particularly after colorectal operations^18–25^. The results of these common laboratory tests in the early period after liver transplantation may be subject to the overlapping effects of patient pre-transplant status, operative injury, allograft function, post-transplant immunosuppression and occurrence of non-infectious complications. Although several reports focused on clinical utility of these inflammatory markers in liver transplant setting, data on their diagnostic accuracy, optimal cut-offs and factors influencing their changes in the first days after liver transplantation in an intensive care unit are scarce^26,27^. As these information are crucial for appropriate interpretation of their results, the aim of this study was (i) to evaluate the clinical applicability of procalcitonin, CRP, and NLR in diagnosis of postoperative infections in the first days after liver transplantation in the intensive care unit and (ii) to assess factors influencing their changes.

## Methods

This was a prospective longitudinal study including 60 consecutive patients after first deceased donor liver transplantation in the Department of General, Transplant and Liver Surgery at the Medical University of Warsaw between November 2015 and April 2016. Inclusion criteria comprised age over 18 years and provision of informed consent. Patients with a history of previous liver transplantation were excluded. All participants provided informed consent prior to enrollment to the study. The study protocol was approved by the institutional review board of the Medical University of Warsaw (KB/193/2015). All methods were performed in accordance with the relevant guidelines and regulations. No organs were procured from prisoners. All organs were procured by the transplant team of the Department of General, Transplant and Liver Surgery of the Medical University of Warsaw.

Bacterial and fungal infections occurring during patient postoperative stay in intensive surgical care unit were the primary end-point. They were defined and classified according to Centers for Disease Control guidelines for diagnosing healthcare-associated infections in the acute care setting^28^. Causative microorganisms, clinical management and outcome were recorded. Severe postoperative complications, defined as grade ≥3 according to Clavien-Dindo classification, were set as a secondary end-point^29^. Early allograft dysfunction was diagnosed using criteria proposed by Olthoff *et al*.^30^. Follow-up for the occurrence of clinical end-points was limited to patient stay in the intensive care unit.

Procalcitonin, CRP, and NLR assessed at 12-hour intervals during patient postoperative stay in the intensive surgical care unit were recorded as primary factors of interest. Postoperative changes of these parameters over the first 5 postoperative days were compared between patients with and without infectious complications, with and without severe postoperative complications, and with and without early allograft dysfunction. Regarding these comparisons, only infections and severe complications occurring during first 5 postoperative days were considered. Irrespective of the presence of significant differences between particular subgroups, a series of additional variables representing CRP, procalcitonin, and NLR changes were created in an exploratory fashion basing on evaluation of their five-day kinetics and assessed as predictors of infections and severe morbidity. Pre-transplant, intraoperative, donor, and postoperative factors were subsequently evaluated for significant influence on postoperative changes of the three inflammation markers. Postoperative laboratory results other than CRP, procalcitonin, and NLR were included in these analyses basing on correlations with these inflammation markers and following transformation to single variables.

The protocol of standard antimicrobial prophylaxis included intravenous piperacillin/tazobactam for three days and fluconazole for seven days. In patients colonized with gram-negative extended-spectrum beta-lactamase producing pathogens, perioperative prophylaxis was based on imipenem/cilastatin and vancomycin. Immediately after the operation patients were transferred to intensive surgical care unit. Immunosuppression protocol was based on combinations of steroids, tacrolimus, and mycophenolate mofetil. Basiliximab was applied for induction immunosuppression in selected patients basing on clinical indications. The choice over use of induction immunosuppression was made at the discretion of the consulting hepatologist, yet generally, at the time of study basiliximab was not preferred in patients with alcoholic liver disease or autoimmune-related liver disease. Peripheral and central catheter blood cultures were routinely performed 24 and 48 hours post transplantation. All grafts were procured from donors after brain death. Transplantations were performed either with piggyback technique and side-to-side caval anastomosis or with conventional technique and two end-to-end caval anastomoses. Temporary extracorporeal veno-venous circulation was used during conventional procedures. Other details on perioperative care and surgical technique were provided elsewhere^31,32^.

Data were presented as medians with interquartile ranges or frequencies with percentages for quantitative and qualitative variables, respectively. General linear models for repeated measurements were used to evaluate postoperative changes of inflammatory markers. Multivariable analyses included only factors with significant intergroup effects on univariable analyses with final model created using backward elimination method (p > 0.05 applied for exclusion of variables). Missing variables were imputed with median values. Logistic regression models were used to evaluate associations between single factors representing CRP, procalcitonin, and NLR changes and occurrence of infections and severe postoperative complications. Receiver operating characteristics analyses were applied for establishment of optimal cut-offs based on Youden index. Sensitivity, specificity, positive predictive value (PPV) and negative predictive value (NPV) were calculated. Correlations between laboratory values in the postoperative period were assessed using Spearman correlation coefficient. Bonferroni correction was applied to manage multiple comparisons in these analyses. Changes of inflammation markers in the postoperative period were presented as least squares means with 95% confidence intervals (95% CIs). Odds ratios (ORs) and areas under the curves (AUCs) were presented with 95% CIs. The level of significance was set to two-sided p < 0.05. All analyses were computed using STATISTICA 13 (Dell Inc., OK, USA) software.

## Results

Basic characteristics of the study cohort are presented in Table 1. Overall, nine patients (15.0%) developed infections during early post-operative intensive care unit stay, including: pneumonia in seven patients (11.7%, two with concomitant secondary bloodstream infection) and other lower respiratory tract infection, urinary tract infection, and surgical site infection, each in one patient. Eight of those nine patients developed infections within the first five postoperative days. Severe postoperative complications occurred in 17 patients (28.3%) and early allograft dysfunction occurred also in 17 patients (28.3%). All severe complications occurred over the first 5 postoperative days and included renal failure requiring renal replacement therapy (n = 5; 8.0%), respiratory failure requiring mechanical ventilation (n = 5; 8.0%), pharyngeal bleeding managed with tamponade (n = 4; 6.6%), intraabdominal bleeding managed surgically (n = 3; 5.0%), circulatory failure (n = 3; 5.0%), hepatic artery thrombosis managed with retransplantation (n = 2; 3.3%), pneumothorax managed with thoracic drainage (n = 1; 1.6%), hydrothorax managed with pleurocentesis (n = 1; 1.6%), primary graft non-function (n = 1; 1.6%), and outflow obstruction syndrome requiring rentransplantation (n = 1; 1.6%).

**Table 1:** Baseline characteristics of 60 patients included in the study.

Variables	n (%) or median (IQR)
**Recipients at the time of transplantation**	
Male sex	35 (58.3%)
Age (years)	50 (34–58)
BMI (kg/m^2^)	24.9 (21.1–27.2)
MELD	14.5 (9.0–21.5)
Child-Turcotte-Pugh classification:	
A	23 (38.3%)
B	22 (36.7%)
C	15 (25.0%)
HCV infection	20 (33.3%)
HBV infection	4 (6.7%)
Alcoholic liver disease	5 (8.3%)
Hepatocellular carcinoma	10 (16.7%)
Autoimmune disease (PSC/PBC/AIH)	17 (28.3%)
White blood count (10^3^/µL)	5.85 (4.32–8.52)
NLR	2.87 (1.87–4.80)
Red blood count (10^6^/µL)	3.80 (2.98–4.32)
Neutrophils (10^3^/µL)	3.51 (2.38–5.14)
Lymphocytes (10^3^/µL)	1.10 (0.78–2.01)
Platelets (10^3^/µL)	116.0 (59.5–187.5)
Hemoglobin concentration (g/dL)	11.70 (9.85–12.95)
Bilirubin concentration (mg/dL)	2.21 (0.87–9.30)
Albumin concentration (g/dL)	3.40 (2.85–4.20)
Creatinine concentration (mg/dL)	0.89 (0.71–1.13)
Urea concentration (mg/dL)	31.5 (24.0–53.0)
Alanine transaminase activity (U/L)	46.0 (31.5–71.5)
Aspartate transaminase activity (U/L)	63.0 (41.0–112.0)
Gamma-glutamyl-transpeptidase activity (U/L)	83.5 (34.0–149.0)
Alkaline phosphatase activity (U/L)	129.0 (108.0–194.0)
INR	1.34 (1.15–1.60)
Lactate concentration (mmol/L)	1.2 (1.0–1.7)
CRP concentration (mg/L)	5.1 (1.75–21.9)
Procalcitonin concentration (ng/mL)	0.08 (0.05–0.44)
**Transplantation**	
Total ischemic time (hours)	8.5 (7.0–10.0)
Cold ischemic time (hours)	7.25 (5.9–8.9)
Warm ischemic time (minutes)	49 (40–60)
Duration of anhepatic phase (minutes)	91 (67–118)
Operative time (hours)	6.58 (5.5–7.2)
*Piggyback* technique	54 (90.0%)
Biliary anastomosis:	
Duct-to-duct	50 (83.3%)
Hepaticojejunostomy	10 (16.7%)
Packed red blood cells transfusions (units)	4 (2–6)
Fresh frozen plasma transfusions (units)	4 (0–8)
Intraoperative dialysis	6 (10.0%)
**Immunosuppression**	
Tacrolimus	58 (96.7%)
Mycophenolate mofetil	54 (90.0%)
Basiliximab	35 (58.3%)
**Donors**	
Male sex	43 (71.7%)
Age (years)	49.0 (31.5–59.0)
CRP concentration (mg/L)	178.0 (55.8–268.0)
Infection	5 (8.3%)

IQR – interquartile range; BMI – body mass index; MELD – model for end-stage liver disease; HCV – hepatitis C virus; HBV – hepatitis B virus; PSC – primary sclerosing cholangitis; PBC – primary biliary cirrhosis; AIH – autoimmune hepatitis; NLR – neutrophil-to-lymphocyte ratio; CRP – C-reactive protein.

**Figure 1:**
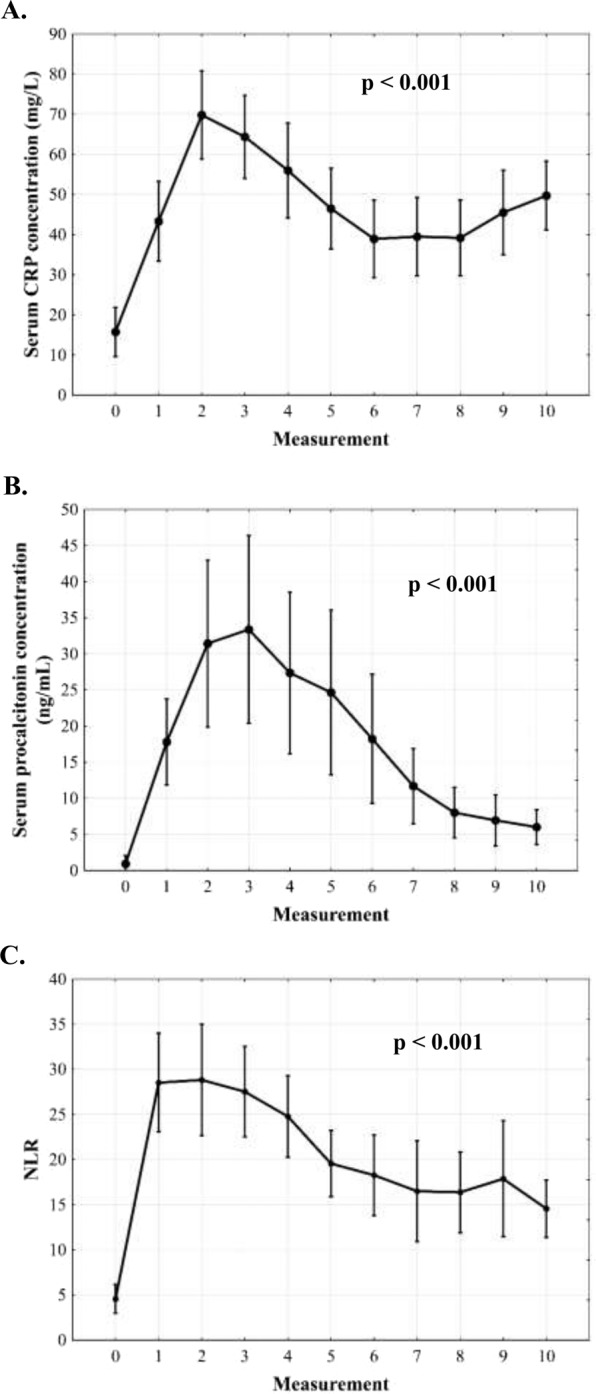
Changes of C-reactive protein (CRP) concentration (**A**), procalcitonin concentration (**B**), and neutrophil-to-lymphocyte ratio (NLR, **C**) over 5 days after liver transplantation. Measurement 0 corresponds to preoperative samples. Measurements 1–10 correspond to subsequent postoperative samples obtained in 12-hour intervals.

**Figure 2:**
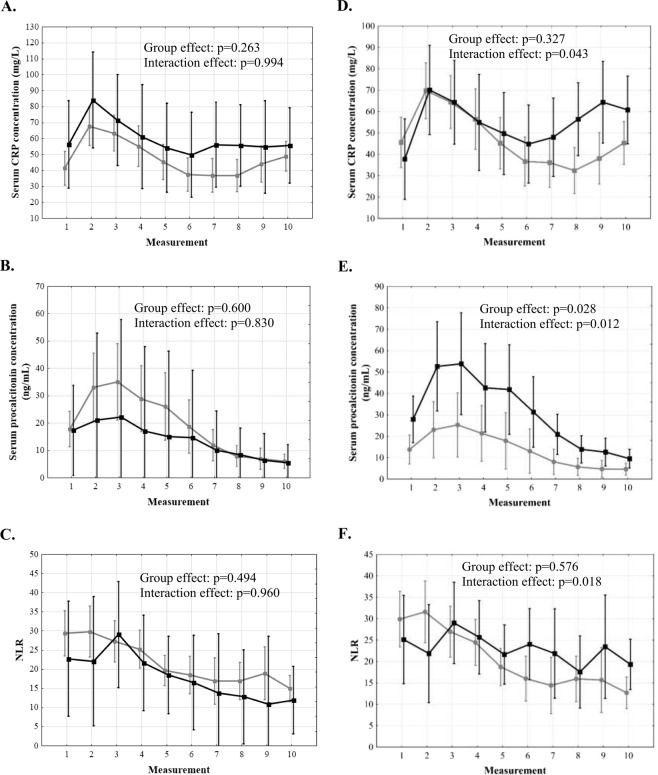
Differences in C-reactive protein (CRP) concentration, procalcitonin concentration, and neutrophil-to-lymphocyte ratio (NLR) over 5 days after liver transplantation between patients with (black) and without (grey) infections (**A**–**C**) and between patients with (black) and without (grey) severe complications (**D**–**F**). Measurements 1–10 correspond to subsequent postoperative samples obtained in 12-hour intervals.

**Figure 3:**
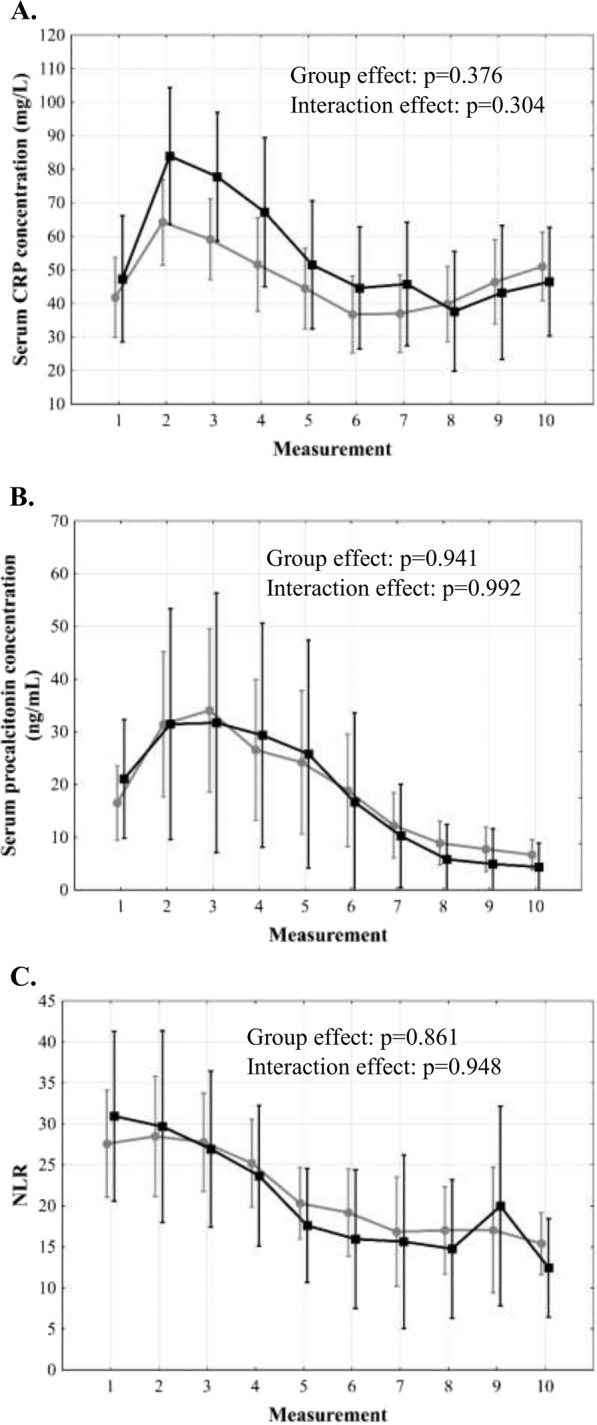
Differences in C-reactive protein (CRP) concentration (**A**), procalcitonin concentration (**B**), and neutrophil-to-lymphocyte ratio (NLR, **C**) over 5 days after liver transplantation between patients with (black) and without (grey) early allograft dysfunction. Measurements 1–10 correspond to subsequent postoperative samples obtained in 12-hour intervals.

There was a sharp rise of CRP in the first 24 hours post-transplantation with subsequent decline and plateau phase after 48 hours (p < 0.001; Fig. 1A). Procalcitonin changes were characterized by a sharp rise over the first 36 hours and subsequent decline (p < 0.001; Fig. 1B). NLR increased immediately following transplantation, remained stable for 36–48 hours and then gradually declined (p < 0.001; Fig. 1C). There were no significant differences in CRP, procalcitonin, and NLR over 5 post-transplant days depending on the occurrence of infections (Fig. 2A–C). Patients with severe postoperative complications exhibited second increase of CRP beyond 48 hours (interaction effect: p = 0.043; Fig. 2D), higher procalcitonin with more dynamic changes in the entire analyzed period (group effect: p = 0.028; interaction effect: p = 0.012; Fig. 2E), and a lack of NLR decline (interaction effect: p = 0.018; Fig. 2F). No significant differences in these inflammation markers were observed depending on the occurrence of EAD (Fig. 3).

**Figure 4:**
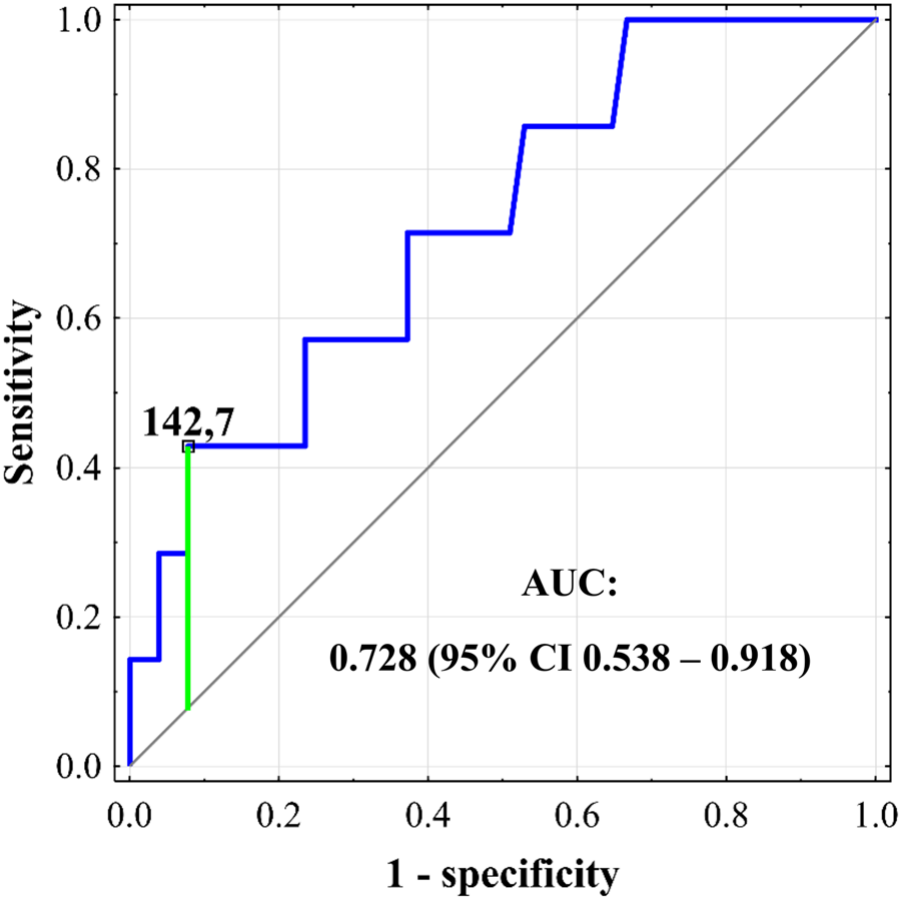
Receiver operating characteristics curve for prediction of infections after liver transplantation based on peak C-reactive protein concentration beyond first 48 postoperative hours. Cut-off is provided in mg/L. Area under the curve (AUC) is presented with 95% confidence interval (95% CI).

A series of additional variables representing CRP, procalcitonin, and NLR values was created. Of those representing CRP changes, only peak CRP concentration beyond first 48 postoperative hours was significantly associated with infections (p = 0.038; Table 2). The optimal cut-off was 142.7 mg/L with an AUC of 0.728 (95% CI 0.538–0.918), sensitivity of 42.9%, specificity of 92.2%, PPV of 42.9% and NPV of 92.2% (Fig. 4). None of procalcitonin- or NLR-based variables was significantly associated with the occurrence of infections.

**Table 2:** Associations between inflammation markers and development of infections after liver transplantation.

Variable	OR	95% CI	p
CRP_max_	1.08	0.97–1.20	0.158
CRP_max5–10_	1.13	1.01–1.26	0.038
CRP_max1–4_	1.04	0.92–1.18	0.499
CRP_Δmax5–10/max1–4_	1.09	0.96–1.26	0.184
PCT_max_	1.01	0.94–1.07	0.865
PCT_max6–10_	1.02	0.92–1.12	0.798
PCT_max1–5_	1.01	0.94–1.07	0.865
PCT_Δmax6–10/max1–5_	0.97	0.85–1.10	0.623
NLR_max_	0.99	0.96–1.02	0.408
NLR_max5–10_	0.99	0.96–1.03	0.693
NLR_max1–4_	0.99	0.96–1.02	0.545
NLR_Δmax5–10/max1–4_	1.00	0.96–1.03	0.772

Odds ratios are given per: 10 mg/L increase for CRP; 5 ng/ml increase for procalcitonin; and 1 increase for NLR. OR – odds ratio; 95% CI – 95% confidence interval; CRP – C-reactive protein; PCT – procalcitonin; NLR – neutrophil-to-lymphocyte ratio.

CRPmax – peak CRP concentration in all measurements.

CRPmax5–10 – peak CRP concentration in measurements 5–10 (beyond first 48 post-transplant hours).

CRPmax1–4 – peak CRP concentration in measurements 1–4 (over first 48 post-transplant hours).

CRPΔmax5–10/max1–4 – difference between peak CRP concentration in measurements 5–10 (beyond first 48 post-transplant hours) and peak CRP concentration in measurements 1–4 (over first 48 post-transplant hours).

PCTmax – peak procalcitonin concentration in all measurements.

PCTmax6–10 – peak procalcitonin concentration in measurements 6–10 (beyond first 60 post-transplant hours).

PCTmax1–5 – peak procalcitonin concentration in measurements 1–5 (over first 60 post-transplant hours).

PCTΔmax6–10/max1–5 – difference between peak procalcitonin concentration in measurements 6–10 (beyond first 60 post-transplant hours) and peak procalcitonin concentration in measurements 1–5 (over first 60 post-transplant hours).

NLRmax – peak NLR in all measurements.

NLRmax5–10 – peak NLR in measurements 5–10 (beyond first 48 post-transplant hours).

NLRmax1–4 – peak NLR in measurements 1–4 (over first 48 post-transplant hours).

NLRΔmax5–10/max1–4 – difference between peak NLR in measurements 5–10 (beyond first 48 post-transplant hours) and peak NLR in measurements 1–4 (over first 48 post-transplant hours).

**Figure 5:**
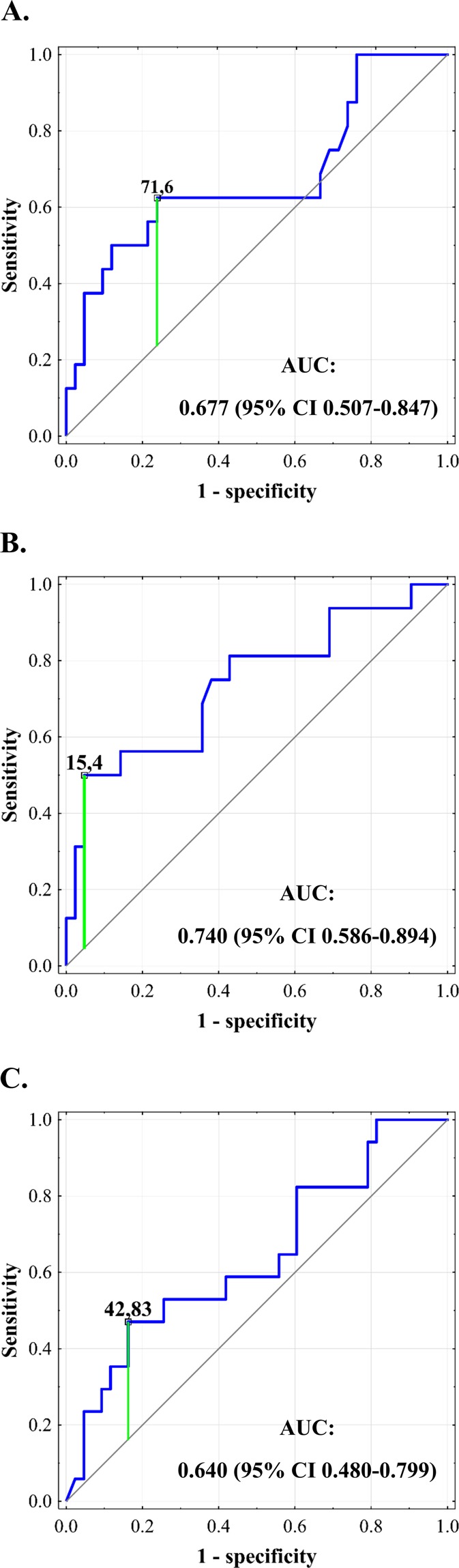
Receiver operating characteristics curves for prediction of severe complications after liver transplantation based on peak C-reactive protein concentration beyond first 48 postoperative hours (**A**), difference between peak C-reactive protein concentration beyond and within first 48 hours (**B**), and peak procalcitonin concentration over first 60 hours (**C**). Cut-offs for C-reactive protein are provided in mg/L and cut-off for procalcitonin is provided in ng/mL. Areas under the curve (AUCs) are presented with 95% confidence intervals (95% CIs).

Occurrence of severe postoperative complications was significantly associated with peak CRP beyond first 48 hours (p = 0.016), difference between peak CRP beyond and within first 48 hours (p = 0.007), and peak procalcitonin over first 60 hours (p = 0.050; Table 3). As peak procalcitonin in general was identical to peak procalcitonin within first 60 hours, it was disregarded in further analyses. The optimal cut-off for peak CRP beyond first 48 hours was 71.6 mg/L with an AUC of 0.677 (95% CI 0.507–0.847), sensitivity of 62.5%, specificity of 76.2%, PPV of 50.0% and NPV of 84.2% (Fig. 5A). The optimal cut-off for difference between peak CRP beyond and within first 48 hours was 15.4 mg/L with an AUC of 0.740 (95% CI 0.586–0.894), sensitivity of 50.0%, specificity of 95.2%, PPV of 80.0% and NPV of 83.3% (Fig. 5B). The optimal cut-off for peak procalcitonin over first 60 hours was 42.8 ng/mL with an AUC of 0.640 (95% CI 0.480–0.799), sensitivity of 47.1%, specificity of 83.7%, PPV of 53.3% and NPV of 80.0% (Fig. 5C).

**Table 3:** Associations between inflammation markers and development of severe complications after liver transplantation.

Variable	OR	95% CI	p
CRP_max_	1.06	0.97–1.16	0.181
CRP_max5–10_	1.13	1.02–1.24	0.016
CRP_max1–4_	0.97	0.87–1.09	0.638
CRP_Δmax5–10/max1–4_	1.26	1.06–1.49	0.007
PCT_max_	1.05	1.00–1.11	0.050
PCT_max6–10_	1.07	0.99–1.16	0.116
PCT_max1–5_	1.05	1.00–1.11	0.050
PCT_Δmax6–10/max1–5_	0.90	0.80–1.01	0.079
NLR_max_	1.00	0.98–1.02	0.949
NLR_max5–10_	1.01	0.99–1.03	0.331
NLR_max1–4_	1.00	0.98–1.02	0.943
NLR_Δmax5–10/max1–4_	1.01	0.99–1.04	0.263

Odds ratios are given per: 10 mg/L increase for CRP; 5 ng/ml increase for procalcitonin; and 1 increase for NLR. OR – odds ratio; 95% CI – 95% confidence interval; CRP – C-reactive protein; PCT – procalcitonin; NLR – neutrophil-to-lymphocyte ratio.

CRPmax – peak CRP concentration in all measurements.

CRPmax5–10 – peak CRP concentration in measurements 5–10 (beyond first 48 post-transplant hours).

CRPmax1–4 – peak CRP concentration in measurements 1–4 (over first 48 post-transplant hours).

CRPΔmax5–10/max1–4 – difference between peak CRP concentration in measurements 5–10 (beyond first 48 post-transplant hours) and peak CRP concentration in measurements 1–4 (over first 48 post-transplant hours).

PCTmax – peak procalcitonin concentration in all measurements.

PCTmax6–10 – peak procalcitonin concentration in measurements 6–10 (beyond first 60 post-transplant hours).

PCTmax1–5 – peak procalcitonin concentration in measurements 1–5 (over first 60 post-transplant hours).

PCTΔmax6–10/max1–5 – difference between peak procalcitonin concentration in measurements 6–10 (beyond first 60 post-transplant hours) and peak procalcitonin concentration in measurements 1–5 (over first 60 post-transplant hours).

NLRmax – peak NLR in all measurements.

NLRmax5–10 – peak NLR in measurements 5–10 (beyond first 48 post-transplant hours).

NLRmax1–4 – peak NLR in measurements 1–4 (over first 48 post-transplant hours).

NLRΔmax5–10/max1–4 – difference between peak NLR in measurements 5–10 (beyond first 48 post-transplant hours) and peak NLR in measurements 1–4 (over first 48 post-transplant hours).

The only factor associated with CRP changes was the presence of alcoholic liver disease (p = 0.038; Table 4). Factors independently associated with procalcitonin changes comprised recipient body mass index (p = 0.049), intraoperative packed red blood cells transfusions (p = 0.002), peak creatinine concentration on post-transplant days 2–5 (p = 0.034), intraoperative dialysis (p = 0.011), and donor infection (p < 0.001). Factors independently associated with NLR changes comprised duration of anhepatic phase (p < 0.001), peak alanine transaminase activity on day two (p < 0.001), and presence of autoimmune disease as indication for transplantation (p = 0.011).

**Table 4:** Multivariable analyses of factors independently associated with C-reactive protein concentration, procalcitonin concentration, and neutrophil-to-lymphocyte ratio over 5-day period after liver transplantation.

Factors	Group effect		Interaction effect	
	p	η^2^	p	η^2^
**Factor associated with C-reactive protein concentration**				
Alcoholic liver disease	0.038	0.072234	0.305	0.019928
**Factors independently associated with procalcitonin concentration**				
BMI	0.049	0.071309	<0.001	0.082937
Intraoperative packed red blood cells transfusions	0.002	0.165313	<0.001	0.074209
Postoperative peak creatine concentration (measurements 3–10)	0.034	0.082469	0.001	0.059063
Intraoperative dialysis	0.011	0.115511	<0.001	0.094143
Infection in the donor	<0.001	0.378823	<0.001	0.317042
**Factors independently associated with NLR**				
Duration of anhepatic phase	<0.001	0.259049	<0.001	0.262757
Postoperative peak alanine transaminase activity (measurements 3 and 4)	<0.001	0.268152	<0.001	0.079855
Autoimmune disease (PSC/PBC/AIH)	0.011	0.112663	0.026	0.037167

BMI – body mass index; PSC – primary sclerosing cholangitis; PBC – primary biliary cirrhosis; AIH – autoimmune hepatitis; NLR – neutrophil-to-lymphocyte ratio.

## Discussion

Early diagnosis of infections after liver transplantation is a necessary prerequisite for timely initiation of antimicrobial therapy, which increases the odds for favorable outcomes. Both CRP and procalcitonin are used for surveillance of postoperative infections after non-transplant operations. NLR, a well-established marker of systemic inflammatory response, currently being investigated for that purpose. The present study indicates that CRP, procalcitonin, and NLR are not reliable markers for diagnosis of infectious complications in the immediate period after liver transplantation in an intensive care setting, yet CRP may be used for initially excluding the diagnosis of infectious complications.

Of the analyzed inflammatory markers, only peak CRP beyond first 48 postoperative hours was found to be significantly associated with infections. However, sensitivity and PPV rates below 50% indicate that it cannot be used for accurate diagnosis and its role is rather limited to identification of high risk patients. This seems to be its universal shortcoming, given that low PPVs are commonly reported for CRP in surveillance of infections also following non-transplant operations^18,21,23^. Due to relatively high NPV, it seems that CRP beyond first 48 hours may be applied for initial exclusion of the diagnosis of infection. This findings partly correspond to the results of studies on patients undergoing non-transplant abdominal operations, in which CRP on postoperative days three, four, and five were all associated with the development of infectious complications. In meta-analysis by Adamina *et al*. involving nearly two thousand patients undergoing various abdominal procedures, the highest AUC for CRP on days 3–5 was 0.76 with the highest specificity, sensitivity, PPV and NPV rates of 76%, 83%, 53% and 86%, respectively^21^. More recent meta-analysis focused on colorectal procedures revealed even higher AUCs for CRP on these days, ranging from 0.79 to 0.87 with the highest specificity, sensitivity, PPV and NPV rates of 81%, 75%, 24%, and 98%, respectively^18^. Present study indicates that the use of CRP for surveillance of infections in the very early period after liver transplantation is characterized by similar AUC, similar specificity and NPV, similar or slightly higher PPV, but remarkably lower sensitivity. Therefore, an increased number of infectious complications may be missed using peak CRP beyond first 48 hours for excluding infections after liver transplantation than after non-transplant abdominal operations. This points towards a rather adjunct role of CRP in excluding the diagnosis of infectious complications early after liver transplantation, which should be done in the context of other factors, comprehensive clinical assessment and type of immunosuppression, as the use of mycophenolate mofetil independently influenced its postoperative course.

Interestingly, procalcitonin was not found useful for surveillance of infections immediately after liver transplantation in an intensive care unit. Further, procalcitonin was remarkably, yet non-significantly lower in patients experiencing infectious episodes than in those without infections, particularly in the first two post-transplant days. This is contrary to the findings of studies performed in patients undergoing non-transplant operations, in whom peak procalcitonin in the early postoperative period was significantly associated with the development of infectious complications^18–20,23^. One of the potential reasons for a lack of clinical utility of procalcitonin for infection surveillance early after liver transplantation includes the multifactorial genesis of its post-transplant course. Factors influencing postoperative procalcitonin changes comprised those related to the preoperative recipient status (body mass index), intraoperative course (transfusions, the need for intraoperative dialysis), post-operative course (creatinine), and donor microbiological status. Procalcitonin may, however, be used to predict development of severe postoperative complications considering remarkable differences observed as early as on the first postoperative measurement, performed within first 12 postoperative hours. This confirms previous findings from non-transplant setting for the field of liver transplantation^33–35^. Nevertheless, these differences also seem to derive from perioperative factors influencing its postoperative changes, such as the need for intraoperative dialysis or transfusion requirement, as they indicate altered course of the transplantation or more severely ill recipient. While both peak CRP beyond first 48 hours and difference between peak CRP beyond and within first 48 hours were also significantly associated with severe morbidity with higher specificity and PPV of the latter, earlier timing of peak concentration seems to be the most clinically important advantage of procalcitonin. Notably, the postoperative course of inflammation markers was not dependent on liver function.

The present study provides no evidence for clinical utility of NLR for surveillance of post-transplant infections. Although analysis of postoperative changes of NLR in patients with and without severe complications revealed a lack of decreasing slope in the former, the results of further analyses did not prove its clinical applicability and thus, this observation seems only of scientific importance. As the factors independently associated with NLR changes comprised both duration of anhepatic phase and transaminase activity, it seems to be largely driven by ischemia-reperfusion injury. This novel finding is partly consistent with recent reports on the corresponding association between increased NLR and small-for-size syndrome in living donor liver transplantations^36,37^. Significant influence of the presence of autoimmune disease on NLR was reported previously^38^.

Few studies on the relevance of inflammation markers in the early postoperative period were performed in a population of liver transplant recipients. In a study by Perrakis *et al*., fist peak of procalcitonin was not predictive of complicated postoperative course and occurrence of second peak was significantly associated both with infectious and non-infectious complications^39^. Conversely, van den Broek *et al*. revealed significant association between first peak procalcitonin and development of clinically significant infections in an intensive care unit in a timeframe similar to that utilized in the present study, yet it was not an independent predictor of infections, in contrast to peak CRP^26^. According to a report by Eyraud *et al*., post-transplant procalcitonin was remarkably influenced by presence of infection in the donor and was not predictive of infections in the recipients, similar to the present study^40^. No differences in procalcitonin were also reported in the first week following pediatric liver transplantations depending on development of infections^41^. Occurrence of bacteriemia was found by Kaido *et al*. to be associated with differences in procalcitonin after liver transplantation, yet only beyond fifth postoperative day^27^. CRP cut-off of 52 mg/L at 72 hours post-transplantation was proposed for initiation of antibiotic therapy and supported by a PPV of more than 70% with respect to infections^42^. However, despite nearly three-fold higher threshold for peak CRP established in the present study, PPV was only slightly above 40% thus indicating its far lower predictive ability than that reported previously.

The present study has several limitations. First, it is limited by the number of patients which increases the odds for type II error. However, multiple clinically and statistically significant associations of infections, severe morbidity and other variables with the course of inflammation markers were identified. Notably, the observation period was limited to the earliest time after liver transplantation, which is reflected by predominant occurrence of respiratory tract infections and relatively low overall infection rate. Therefore, the results of this study may be interpreted only with respect to the period of first days after transplantation, which is consistent with the aims. Further, the follow-up period was also limited to patient stay in the intensive care unit, which is dependent on departments practice pattern. In this study, patients were discharged from the intensive care unit only in case of uneventful clinical course and consistent improvement of organ function. As the analyses were limited to immediate postoperative period and performed on a limited number of patients, we were unable to provide information on the association between the type of immunosuppression and the course of inflammatory markers in the postoperative period. However, such analyses would be subject to bias related to potential changes in immunosuppression in patients with suspected occurrence of infectious complications. Finally, peak procalcitonin and peak CRP concentrations beyond 48 hours did not always precede actual occurrence of severe complications and infections. However, this does not seem to preclude their use as additional markers in the decision-making process in doubtful cases.

In conclusion, CRP beyond first 48 postoperative hours may be used for initial exclusion of the presence of infectious complications after deceased donor liver transplantation. Both procalcitonin and NLR do not seem clinically useful for infection surveillance immediately after the procedure. Peak procalcitonin may be used for prediction of severe morbidity. CRP, procalcitonin, and NLR are not related to allograft function.

## Data Availability

The datasets generated during and/or analysed during the current study are available from the corresponding author on reasonable request.
